# Achilles Tendon Rupture Following Extracorporeal Shockwave Therapy for Chronic Tendinopathy Despite Symptomatic Resolution: A Case Report

**DOI:** 10.7759/cureus.115484

**Published:** 2026-08-31

**Authors:** Glenn Briffa, Tiziana Mifsud, Francesca Borg, Mark-Anthony Scicluna, Kirill Micallef Stafrace

**Affiliations:** 1 Surgery, Mater Dei Hospital, Msida, MLT; 2 Podiatry, Faculty of Health Sciences, University of Malta, Msida, MLT; 3 Medical Imaging, Mater Dei Hospital, Msida, MLT; 4 General Medicine, Mater Dei Hospital, Msida, MLT; 5 Sports Medicine, Triagon Academy, Marsa, MLT

**Keywords:** achilles tendinopathy, achilles tendon rupture, case report, extracorporeal shockwave therapy, tendon biomechanics, tendon degeneration

## Abstract

Extracorporeal shockwave therapy (ESWT) is widely used in the management of chronic Achilles tendinopathy (AT) and is associated with improved pain and functional outcomes. However, symptomatic recovery may not necessarily reflect restoration of tendon structural integrity or mechanical resilience. We report the case of a 61-year-old active male with a ten-year history of chronic mid-portion AT refractory to conservative management who underwent six sessions of radial ESWT, resulting in complete pain resolution and return to unrestricted sporting activity. Six months after completing treatment, he sustained an acute Achilles tendon rupture following a low-energy mechanism while stepping off a pavement. Clinical examination and point-of-care ultrasound confirmed tendon discontinuity with apposable tendon ends. The patient underwent non-operative management with equinus immobilization and progressive rehabilitation. Follow-up ultrasound demonstrated partial tendon healing, although persistent pain and functional limitation remained at the latest follow-up. This case highlights the potential dissociation between symptomatic improvement and structural tendon recovery in chronic AT. Although no causal relationship between ESWT and tendon rupture can be inferred, symptomatic improvement may permit return to higher loading demands despite persistent underlying tendon degeneration. Clinicians should recognize that symptom resolution does not necessarily indicate restoration of tendon mechanical resilience and should consider appropriate counseling and graduated return-to-loading strategies following successful ESWT.

## Introduction

Achilles tendinopathy (AT) is a common degenerative tendon disorder characterized by collagen disorganization, tendon thickening, altered extracellular matrix composition, and progressive reduction in mechanical resilience [1]. Contemporary understanding recognizes tendinopathy as a failed healing response rather than a purely inflammatory process, with structural degeneration occurring along a continuum of tendon pathology [2,3]. Importantly, pain, function, and tendon structure do not consistently correlate, and symptomatic improvement may occur despite persistence of underlying degenerative change [2,4,5].

Extracorporeal shockwave therapy (ESWT) is a widely used non-operative treatment for chronic mid-portion AT in patients with persistent symptoms despite conservative management [6]. While ESWT is supported by evidence of symptomatic and functional benefit, the extent to which it restores underlying tendon biomechanical integrity remains uncertain [5,7].

Achilles tendon rupture is a recognized complication of chronic degenerative tendinopathy, particularly within the hypovascular mid-portion of the tendon [1,8,9]. Rupture occurring after apparent clinical recovery may therefore have important implications for patient counseling, return-to-loading strategies, and interpretation of treatment success in chronic tendinopathy.

Rare reports have described Achilles tendon rupture temporally associated with ESWT; however, these have generally occurred during or shortly after treatment in patients with additional predisposing factors, such as pre-existing calcific tendinopathy or prior tendon surgery [10,11]. To our knowledge, delayed rupture occurring after complete symptomatic resolution and unrestricted return to sporting activity has not previously been reported.

We present a case of delayed Achilles tendon rupture following apparent symptomatic recovery after ESWT in a patient with longstanding chronic mid-portion AT. This temporal sequence does not, in itself, establish a causal relationship between ESWT and rupture; rather, the case highlights the potential dissociation between clinical improvement and underlying tendon structural integrity in chronic degenerative tendinopathy.

This case report has been reported in line with the Surgical CAse REport (SCARE) 2023 criteria [12]. This work has not been previously presented in any form.

## Case presentation

A 61-year-old active male presented in October 2025 with acute-onset right posterior ankle pain and functional impairment following a minor mechanical event. The patient reported stepping down from a pavement and immediately experiencing a sensation of giving way in the posterior ankle associated with an inability to walk normally or actively plantarflex. Immediate swelling and bruising developed, and he presented for assessment several days later. No audible snap was reported.

The patient had a ten-year history of symptomatic mid-portion, non-insertional AT with symptoms initially beginning in 2015. Ultrasound imaging performed in 2015 confirmed chronic mid-portion AT, demonstrating tendon thickening and degenerative change without evidence of partial or complete tear (Figure 1). Over the subsequent decade, symptoms fluctuated considerably despite prolonged conservative management, including physiotherapy, activity modification, and intermittent use of multimodal analgesia, including short courses of non-steroidal anti-inflammatory medication. Multiple periods of significant pain and limitation of sporting and daily activities were reported, particularly with running. No corticosteroid or other infiltrative treatments had been performed.

**Figure 1:**
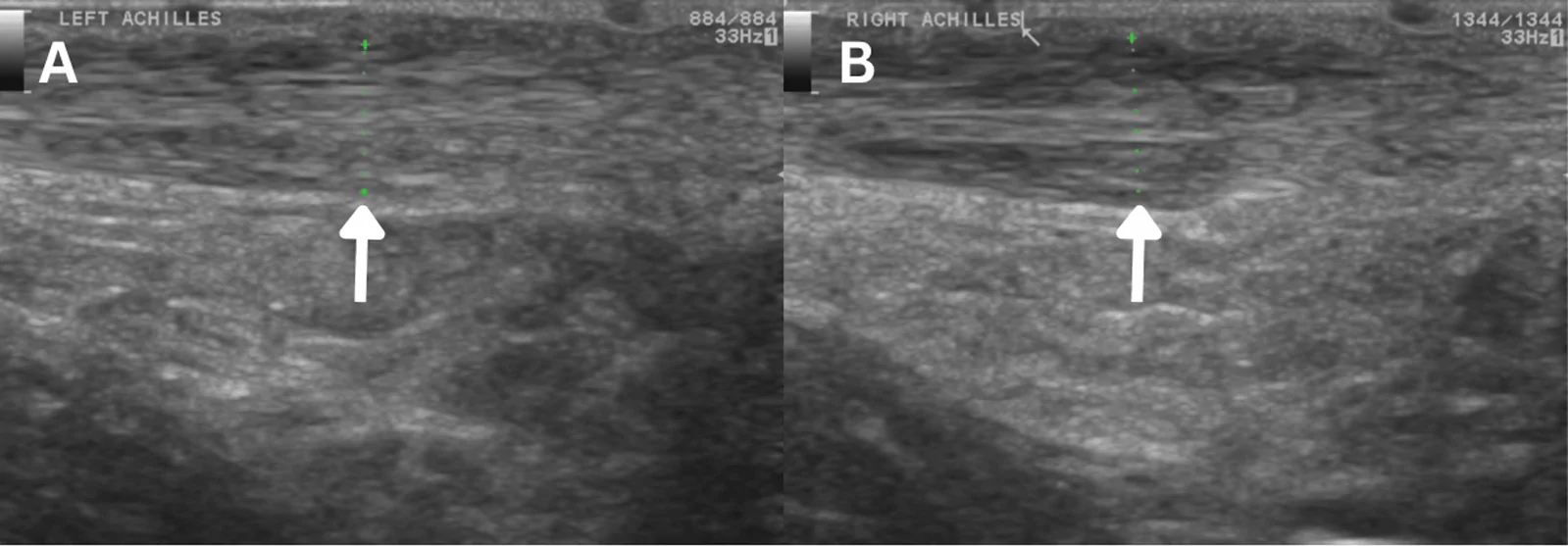
Bilateral chronic Achilles tendinopathy on pre-treatment ultrasound. Longitudinal ultrasound images of the left and right Achilles tendons obtained prior to extracorporeal shockwave therapy demonstrating mid-portion tendon thickening and loss of normal fibrillar architecture consistent with chronic Achilles tendinopathy, without evidence of partial or complete tendon tear. A: left Achilles tendon, with the arrow indicating the diseased tendon (maximum thickness 0.65 cm); B: right Achilles tendon, with the arrow indicating the diseased tendon (maximum thickness 0.76 cm).

Between November 2024 and April 2025, the patient underwent ESWT administered by a sports and exercise medicine specialist using a radial shockwave device (Zimmer EnPuls 2.0; Zimmer MedizinSysteme GmbH, Neu-Ulm, Germany). A total of six treatment sessions were delivered at weekly to biweekly intervals. Sessions one to five consisted of bilateral radial ESWT delivered at an intensity level of 90 mJ (device-specific measure), a frequency of 14 Hz, and 2500 shocks per session. The final session was delivered at the same intensity and frequency with an increased dose of 4000 shocks bilaterally. At review in April 2025, the patient reported complete resolution of pain with a return to normal sporting and daily activities without stiffness or functional limitation and expressed high satisfaction with the treatment outcome. The clinical timeline is summarized in Figure 2.

**Figure 2:**
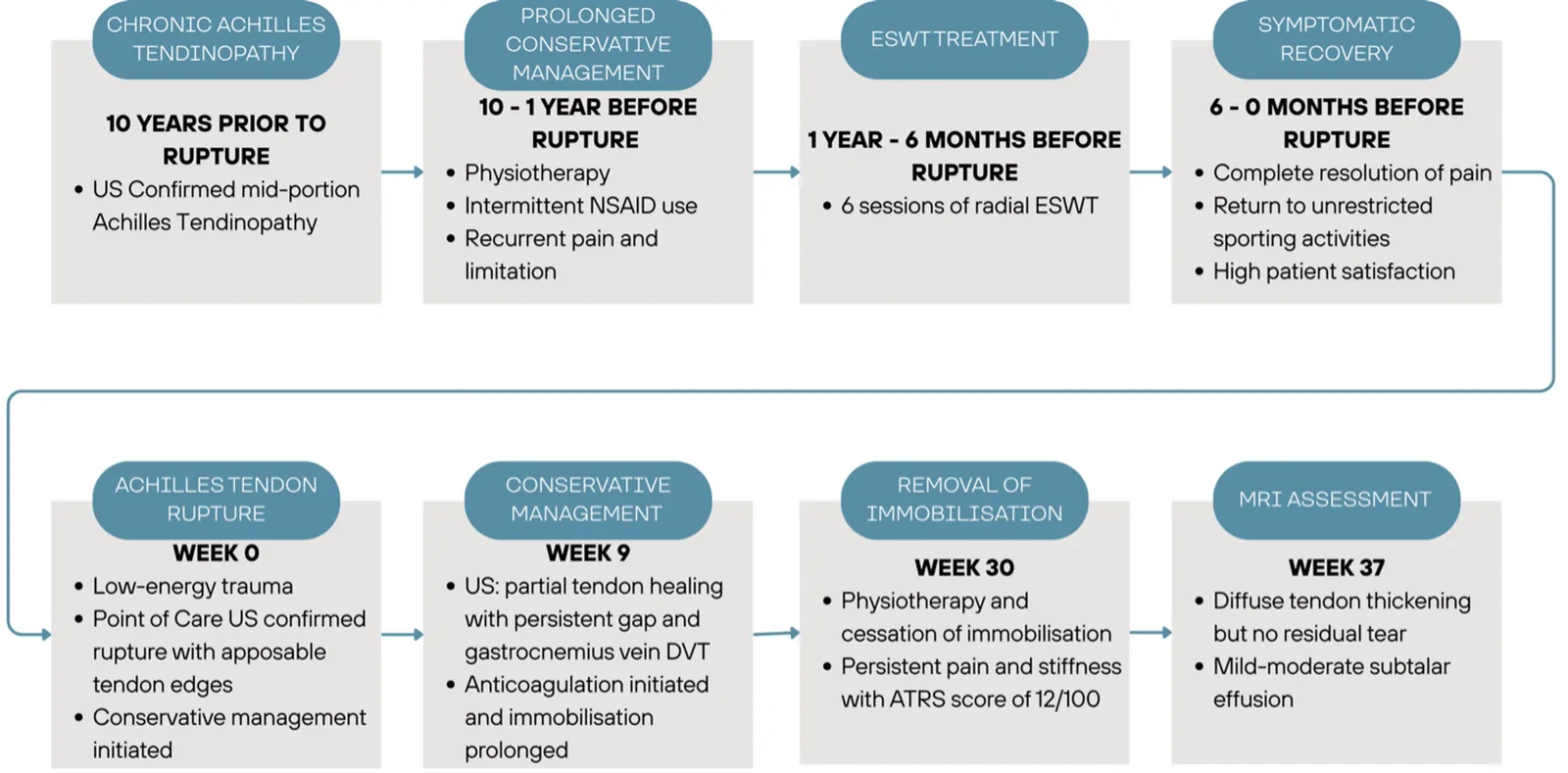
Timeline of clinical course. Pre-rupture history (upper row) is expressed in years before the acute Achilles tendon rupture; the post-rupture course (lower row) is expressed in weeks from the day of rupture (Week 0), through conservative management, removal of immobilization, and MRI assessment at the latest follow-up. NSAID: non-steroidal anti-inflammatory drugs; ESWT: extracorporeal shockwave therapy; DVT: deep vein thrombosis; ATRS: Achilles Tendon Total Rupture Score

Six months after completion of ESWT, the patient sustained the acute injury described above. Medical history was significant for hypertension treated with enalapril 5 mg daily and psoriatic arthritis. He was a non-smoker with normal body mass index and no history of diabetes mellitus, statin use, systemic corticosteroid therapy, fluoroquinolone exposure, prior tendon rupture, or previous local steroid injection. Clinical examination demonstrated swelling along the right Achilles tendon with a palpable gap and a positive Simmonds test, consistent with Achilles tendon rupture. Point-of-care ultrasound confirmed tendon discontinuity with visible separation, but apposable tendon ends during passive plantarflexion. No formal imaging was obtained at the time of acute presentation to avoid delay in management.

Given favorable tendon apposition and patient profile, non-operative management was undertaken according to institutional rehabilitation protocol. The limb was initially immobilized in full equinus using a dorsal slab with non-weight bearing for two weeks, followed by gravity equinus immobilization until four weeks. At four weeks, the patient transitioned to an Aircast walking boot (DJO, LLC, Vista, California, USA) with a 3-cm heel wedge (approximately 22° plantarflexion) with initiation of weight bearing and supervised physiotherapy.

Rehabilitation subsequently progressed with staged reduction of heel wedges, gradual restoration of ankle range of motion, and progressive strengthening exercises according to protocol. Return to sport had initially been anticipated at approximately eight months post-injury.

Follow-up ultrasound imaging performed in December 2025, approximately two months after initiation of conservative management, demonstrated only partial healing of the Achilles tendon with persistent discontinuity of tendon fibers (Figure 3). The examination also identified a gastrocnemius deep vein thrombosis, considered likely secondary to lower limb immobilization and reduced mobility during conservative treatment. Anticoagulation therapy was commenced, and progression of rehabilitation was slowed, with prolongation of walking boot immobilization by an additional month prior to further reduction of heel wedges and advancement of weight-bearing and strengthening exercises.

**Figure 3:**
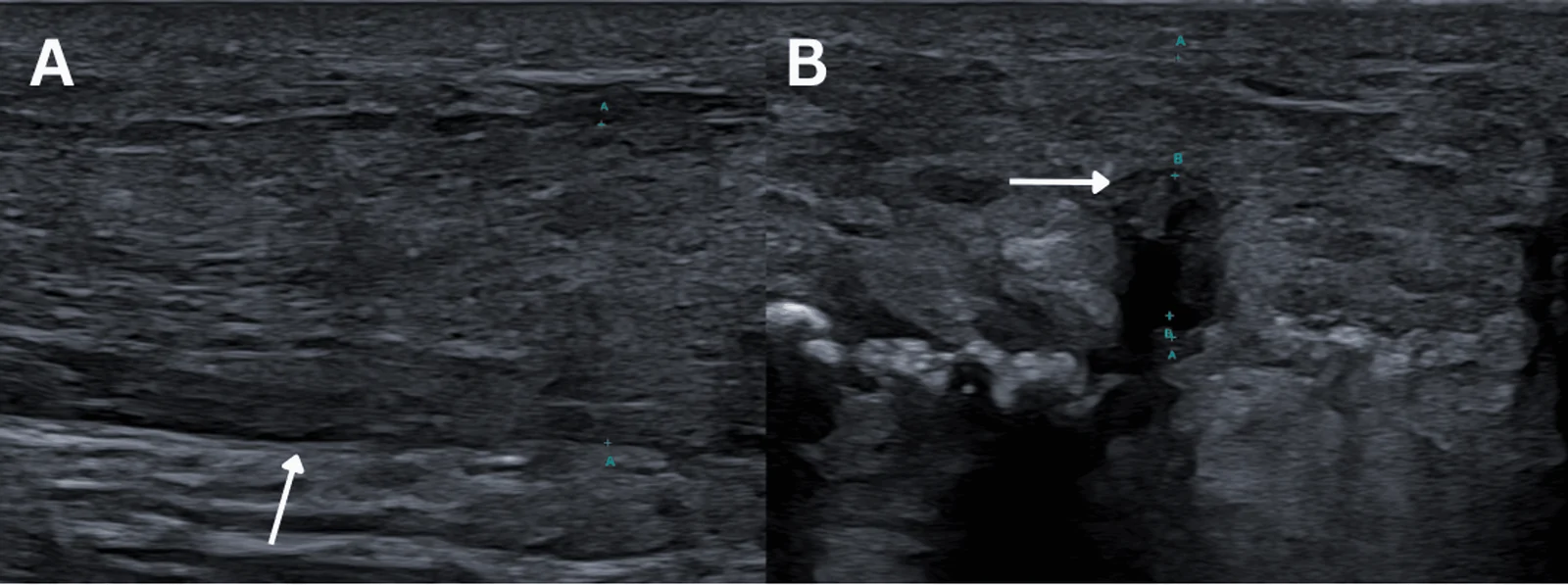
Interval healing of the right Achilles tendon rupture on follow-up ultrasound. Follow-up longitudinal ultrasonography of the right Achilles tendon performed nine weeks after initiation of non-operative management. A: thickened, heterogeneous tissue at the rupture site with disruption of the normal fibrillar architecture (arrow); B: bridging tissue across the rupture site with partial restoration of tendon continuity, although the normal parallel fibrillar architecture remains incompletely restored (arrow).

At the latest follow-up in May 2026, the patient had discontinued immobilization and was undergoing physiotherapy without external support. However, persistent pain and limitation of daily and sporting activities remained present. The Achilles Tendon Total Rupture Score (ATRS) [13] assessment completed in May 2026 demonstrated a score of 12/100 on the affected side. Subsequent MRI performed in June 2026 demonstrated diffuse thickening of the distal 9 cm of the Achilles tendon without evidence of splits or tears, in keeping with diffuse non-insertional tendinopathy, together with a mild-moderate nonspecific ankle-subtalar effusion and a posterior calcaneal spur; the ankle and subtalar joints, plantar fascia, and remaining tendons and ligaments were unremarkable (Figure 4). Long-term clinical outcome remains ongoing.

**Figure 4:**
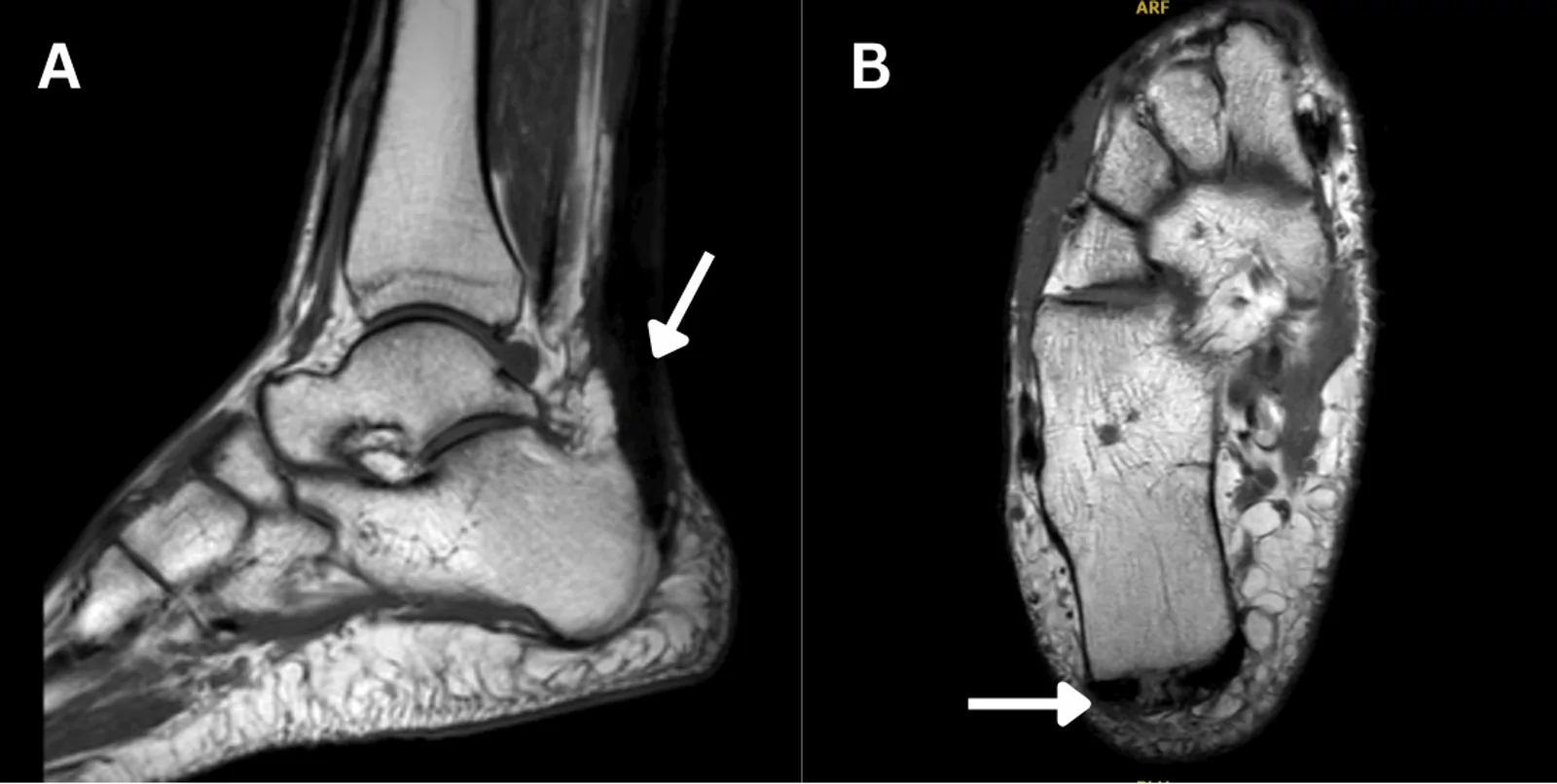
MRI findings of non-insertional Achilles tendinopathy at latest follow-up. Magnetic resonance imaging of the right ankle and hindfoot performed in June 2026. A: sagittal image demonstrating diffuse thickening of the distal Achilles tendon (arrow) over approximately 9 cm, without a discrete split or tear, with a posterior calcaneal spur and a mild-moderate nonspecific ankle-subtalar effusion; B: axial image through the Achilles tendon showing diffuse tendon thickening (arrow) in keeping with non-insertional tendinopathy.

## Discussion

AT represents a complex load-related tendon disorder characterized by collagen disorganization, altered extracellular matrix composition, and impaired mechanical adaptation [1,3]. Contemporary tendon models conceptualize tendinopathy as a continuum in which degeneration and attempted repair coexist, explaining the fluctuating clinical course frequently observed in chronic disease [2,4]. Importantly, structural tendon pathology does not consistently correlate with pain or functional limitation, and imaging abnormalities may persist in both symptomatic and asymptomatic individuals [4,5].

ESWT is widely used in the management of chronic mid-portion AT, particularly in patients who fail structured rehabilitation programs. Systematic review evidence supports improvement in pain and functional outcomes following ESWT in lower limb tendinopathies [6], while randomized and retrospective studies have demonstrated symptomatic and functional benefit in chronic AT [14,15]. Proposed therapeutic mechanisms include neuromodulation, alteration of nociceptive signaling, stimulation of neovascularization, and promotion of tissue remodeling processes [6]. However, evidence demonstrating proportional restoration of tendon biomechanical integrity following symptomatic recovery remains limited [5,7].

The present case is clinically notable because complete symptomatic recovery following ESWT was followed by delayed low-energy Achilles tendon rupture approximately six months later. Prior to rupture, the patient had returned to unrestricted sporting activity without pain or functional limitation despite a decade-long history of chronic non-insertional AT and previously documented structural tendon degeneration on ultrasound imaging. The mechanism of injury was minor, involving a simple step down from a pavement, consistent with recognized patterns of failure in chronically degenerative tendons [8,9].

This case therefore highlights the potential dissociation between symptomatic improvement and underlying tendon resilience in chronic tendinopathy. Contemporary pain models emphasize that tendon pain is influenced not only by tissue structure but also by local nociceptive modulation and broader neurophysiological mechanisms [16]. Symptomatic recovery may therefore reflect improved load tolerance and altered nociceptive processing rather than complete biological restoration of tendon structure or mechanical capacity. This interpretation is supported by prospective ultrasonographic studies demonstrating that structural tendon abnormalities may persist despite substantial clinical improvement [7].

Importantly, this case should not be interpreted as evidence of a causal relationship between ESWT and Achilles tendon rupture. Chronic degenerative tendinopathy itself is a recognized predisposing factor for rupture, with histopathological studies demonstrating substantial pre-existing tendon degeneration in many spontaneous ruptures [8,9,17]. The temporal association observed in this case is therefore more likely reflective of progression of underlying chronic tendon pathology rather than a direct treatment-related complication. The absence of recognized pharmacological risk factors, including corticosteroid exposure or fluoroquinolone use, further supports intrinsic tendon degeneration as the primary underlying contributor.

From a clinical perspective, the case raises important considerations regarding patient counselling and return-to-loading strategies following apparent symptomatic recovery in chronic tendinopathy. Symptom improvement alone may not necessarily indicate restoration of normal tendon mechanical integrity, particularly in longstanding degenerative disease [4,5,7]. Patients returning to higher-demand activity following successful symptomatic treatment may therefore continue to harbor structurally vulnerable tendon tissue despite substantial reduction in pain [1,8,9]. Although no evidence-based protocol currently supports routine structural surveillance following ESWT, clinicians should remain aware of the potential mismatch between clinical recovery and residual tendon pathology when advising progression of loading and return to sport [5,7].

Contemporary evidence supports non-operative management as an effective treatment strategy for selected acute Achilles tendon ruptures when combined with structured functional rehabilitation protocols [18,19]. In the present case, conservative treatment was selected because tendon ends remained apposable during plantarflexion on dynamic ultrasound assessment. However, recovery was complicated by delayed tendon healing and gastrocnemius vein thrombosis, with substantial ongoing functional impairment persisting at the latest follow-up despite rehabilitation.

Limitations 

Several limitations should be acknowledged when interpreting this case. Formal imaging at the time of acute rupture was limited to point-of-care ultrasound, as no MRI or formal radiological ultrasound assessment was obtained during initial presentation to avoid delay in management. Follow-up imaging has also been limited, with the ultrasound demonstrating partial tendon healing having been performed privately and the MRI not obtained until approximately eight months after injury, limiting early structural characterization of the healing tendon.

Furthermore, no structural imaging was performed between completion of ESWT and the subsequent rupture. Consequently, it is not possible to determine whether tendon structural integrity had partially recovered, remained materially unchanged, or progressively deteriorated during the six-month asymptomatic interval preceding rupture.

No validated functional outcome measure such as the ATRS was obtained prior to rupture or before ESWT treatment, limiting objective comparison of functional status over time and quantification of symptomatic improvement following ESWT.

In addition, long-term follow-up remains incomplete, as the patient continues to undergo rehabilitation and further investigation. Final functional and radiological outcomes are therefore not yet available. As a single case report, the findings should be interpreted as hypothesis-generating and cannot establish a causal relationship between ESWT and subsequent Achilles tendon rupture.

## Conclusions

This case highlights that symptomatic recovery following ESWT in chronic AT may not necessarily reflect restoration of normal tendon mechanical integrity. In patients with longstanding degenerative tendon disease, clinical improvement and return to activity may occur despite persistence of structurally vulnerable tendon tissue.

Although no causal relationship between ESWT and tendon rupture can be inferred, and the rupture is more likely attributable to progression of the patient's pre-existing chronic degenerative tendinopathy than to the treatment itself, the case reinforces the importance of appropriate patient counseling and cautious progression of loading following apparent symptomatic recovery. Further prospective research evaluating structural and biomechanical tendon recovery after ESWT may help clarify the relationship between clinical improvement and tendon resilience.
